# A cross-sectional study on colorectal cancer screening knowledge and barriers among university students

**DOI:** 10.1186/s12889-025-22510-z

**Published:** 2025-05-21

**Authors:** Hebatalla Abdelmaksoud Abdelmonsef Ahmed, Bander Saad Albagawi, Amany Hamed AboZayed, Ahmed Yousef, Marzouk M. Marzouk, Ibrahim Naif Alenezi, Abeer A. Almowafy, Hoda Ali Ahmed Shiba

**Affiliations:** 1 Public Health and Community Medicine, Faculty of Medicine, Kafr-Elsheikh University, Kafr-Elsheikh City, Egypt; 2 Medical Surgical Department, College of Nursing, Imam Mohammad Ibn Saud Islamic University, Riyadh, Saudi Arabia; 3 Public Health and Community Medicine, Faculty of Medicine, Cairo University, Cairo, Egypt; 4 Public Health and Community Medicine, Damietta Faculty of Medicine, Al-Azhar University, Damietta, Egypt; 5 Department of Public Health Nursing, College of Nursing, Northern Border University, Arar, Saudi Arabia; 6 International Islamic Institute for Population Studies and Research, Al-Azhar University, Cairo, Egypt; 7 Public Health and Community Medicine, Faculty of Medicine for Girls, Al-Azhar University, Cairo, Egypt

**Keywords:** Colorectal cancer, Screening, Knowledge, University students, Medical faculty, Barriers

## Abstract

**Background:**

Colorectal cancer (CRC) is a significant contributor to cancer-related mortality on a global scale. Timely identification by screening can decrease the morbidity and mortality associated with CRC. This study aimed to evaluate the level of knowledge and perceived barriers related to CRC screening among university students.

**Methods:**

A cross-sectional study was carried out among university students between October 2022 and July 2023. Data were gathered using an online survey that evaluated knowledge of CRC, with a specific emphasis on sociodemographic information, risk factors for CRC, warning signs and symptoms, and the available screening methods.

**Results:**

Out of the 2090 students, 74.8% were female, 54.3% were enrolled in the Faculty of Human Medicine, and 20.4% had a family history of CRC. The general knowledge of CRC and its risk factors was inadequate, as indicated by mean scores of 15.95 ± 6.5 and 3.9 ± 1.95, respectively. A total of 5.3% of the participants had received CRC screening, with colonoscopy being the most frequently employed screening method. In their perspective, the primary obstacles to CRC screening were the absence of endorsement from healthcare professionals, feelings of embarrassment, and apprehension over the outcomes.

**Conclusion:**

The study highlights the need for targeted educational campaigns and interventions to improve CRC awareness among university students and address identified obstacles toward screening. However, findings are limited by self-reported data, which may introduce recall bias and affect generalizability.

**Supplementary Information:**

The online version contains supplementary material available at 10.1186/s12889-025-22510-z.

## Introduction

Colorectal cancer (CRC) refers to cancers that develop in the colon, recto-sigmoid junction, and rectum [1]. CRC is a major public health issue worldwide, characterized by high rates of incidence, prevalence, and fatality. By 2030, the incidence of new cases is expected to rise by 60%, resulting in more than 2.2 million additional cases and 1.1 million premature deaths [2]. The American Cancer Society (2021) reports that the lifetime risk of acquiring CRC in the United States is around 1 in 23 for men and 1 in 25 for women [3]. In Egypt, CRC is a frequent malignancy, accounting for 3.4% of male malignancies and 3% of female malignancies [4]. For the year 2020, CRC ranked as the ninth most prevalent cancer in Egypt, with around 4942 newly diagnosed cases and 2717 fatalities [5]. These elevated rates may be attributed to the increasing age of the population, evolving lifestyle and nutritional patterns, insufficient public knowledge, and restricted availability of screening and treatment services [6]. CRC is a complex systemic illness affected by several risk factors, such as age, genetics, lifestyle, and environmental conditions. Nevertheless, age is a highly influential determinant for CRC, with a rising occurrence of CRC in younger persons [7]. Notably, Egypt, Saudi Arabia, the Philippines, and Iran had CRC incidence rates in young adults under the age of 40 of 38%, 21%, 17%, and 15–35%, respectively [8].

Minimizing the morbidity and mortality linked to CRC requires the implementation of preventive measures and early identification. The World Health Organization (WHO) has classified 40% of malignancies as preventable and 40% as curable if identified at an early stage [9]. The most effective screening methods for CRC are yearly fecal immunochemical testing (FIT), adaptive sigmoidoscopy every decade with an annual FIT test, or a colonoscopy every decade [10]. Nevertheless, Egypt has limited resources for CRC diagnosis and does not have any ongoing screening initiatives at the national level [8]. Consequently, CRC is detected at later stages among younger Egyptians, resulting in a median overall survival of about two years for CRC patients in Egypt. Therefore, it is essential to promote knowledge of risk factors, screening methods, and the significance of prevention [11].

Noteworthy, significant transformations have affected education and societal perceptions of universities. Modern universities are now viewed as active contributors to community development, prompting them to embrace their role in addressing current challenges [8]. Colleges foster social responsibility that involves advancing society through education, research, and technology adaptation by integrating community service into their mandates [12]. From a student’s perspective, community service is either a mandatory obligation or a response to others’ needs [13]. Despite that university students participate in volunteer work that allows them to gain knowledge, they often have lower awareness of CRC screening and prevention [14]. It was demonstrated that there was a lack of awareness of CRC screening among university students [15]. Therefore, it is necessary to enhance understanding and consciousness of CRC screening through educational programming, especially if initiated at the academic degree level [16, 17]. However, only a limited number of research have investigated the knowledge, practice, and perceived barriers to CRC, with no previous studies focused on university students in Egypt. Addressing these gaps is essential to inform future educational interventions and public health strategies at the national level. University students, in particular, represent a key demographic for targeted health education programs due to their potential to act as future advocates for health promotion. Hence, this study aims to evaluate the level of knowledge, practice, and perceived barriers related to CRC screening among university students in Egypt.

## Methods

### Study design & population

an analytical Cross-sectional study was conducted on all Egyptian university students who accepted to participate and enrolled in the academic year (2022–2023). The duration of the study extended from October 2022 to July 2023.

### Data collection tool

An online electronic questionnaire was developed after a literature review to evaluate knowledge of CRC risk factors, symptoms, and signs, as well as accessible screening methods, practice, and perceived barriers related to screening (Supplementary file) [18–22]. Students were given a concise explication of the research and its primary objectives. The questionnaire was initially created in English to enhance the response rate and subsequently translated into Arabic. To ensure translation accuracy, we conducted a Forward-backward Arabic translation of the questionnaire by a bilingual expert. The back-translated version was then compared with the original English one for accuracy. The tools were presented to three professor experts from distinct fields (Public Health & Community Medicine and General Surgery) to evaluate the questionnaire’s validity. Their purpose was to determine the extent to which the questionnaire aligns with the study’s objectives and the intended cohort. To ensure reliability, the questionnaire’s internal consistency was assessed using Cronbach’s Alpha (α = 0.868).

Through the assistance of academic connections and research assistants at all the faculties under study, the final questionnaire was posted and disseminated online between October 7th, 2022, and February 23rd, 2023. As a precaution against redundant responses, the survey was restricted to a single response on Google Forms. To encourage participation and maximize the response rate, reminders were sent periodically, and students were motivated to share the questionnaire with their peers. The response rate was more than 95%.

The questionnaire comprised four domains. The first domain focused on sociodemographic data, including age, sex, faculty, and academic year. The second domain assessed participants’ knowledge about CRC, including its preventive measures and risk factors. The third domain evaluated knowledge about warning signs and symptoms of CRC. Finally, the fourth domain explored awareness of the available screening methods for CRC.

### Scoring system

The knowledge section results were evaluated using Bloom’s cut-off point, with a score of 80 to 100% designated as good, 60 to 79% as moderate, and less than 60% as poor. The response to one question was assigned a score of ‘’1’’ for the accurate answer and ‘’0’’ for the incorrect answer and lack of knowledge of the question item. The cumulative score for the section on knowledge of CRC risk factors varied from 0 to 9 points, the section on knowledge of CRC preventive factors varied from 0 to 5 points, the section on knowledge of CRC warning signs varied from 0 to 7 points, the section on knowledge of CRC screening varied from 0 to 10 points, and the overall knowledge ranged from 0 to 31 points [23].

**A pilot study** was conducted on 34 students, who accounted for 10% of the total sample size, to assess the clarity, feasibility, application, and time needed to complete the questionnaires of the study instruments. Based on the findings of the pilot research, the requisite adjustments and enhancements were implemented before data collection.

**Sample size**: The research sample size was determined using the Epi-info statistical software tool [24]. The parameters employed for the computation of sample size were as follows: The study has a 95% confidence level, an 80% power estimation, an absolute precision of 2.5%, and an expected outcome of 32% [25]. The minimum sample size, as determined by the aforementioned criteria, was 1336 students. Approximately 2090 students were recruited for the study, as the questionnaire was extensively disseminated across several faculties within the universities.

### Ethical considerations

Approval was obtained from the Committee of the Damietta Faculty of Medicine IRB, Al-Azhar University (DFM-IRB 00012367-23-07-016). At the outset of the questionnaire, the research objective was clearly articulated, and students were given the option to either accept or decline participation. The initial inquiry in the questionnaire was designed to ascertain the respondent’s agreement to participate and, if affirmative, their ability to answer the remaining questions. Participants have the option to withdraw from the survey at any point prior to its conclusion. The responses provided by the students were both anonymous and confidential.

### Statistical analysis

The data were analysed using the Statistical Package for the Social Sciences” SPSS 22.0 software (IBM Microsoft). Quantitative data normality was tested using Kolmogorov’s test. Categorical variables were presented using numbers and percentages. The Chi-square test, Fisher’s exact test, and Monte Carlo exact test were employed (if more than 20% of the expected cell value is less than 5). Numerical variables were expressed as means and standard deviations. The Mann-Whitney U-test was employed to compare the numerical values between groups. Simple logistic regression analysis was done to assess the effect of various sociodemographic factors on the study’s outcomes, and the results were tabulated as odds ratio and confidence interval. P-value (< 0.05) was adopted as the level of significance.

## Results

### Demographic and academic characteristics of the studied Egyptian university students

A total number of 2090 university students participated in this study, the majority of the participants were female (74.8%), while males represented only 25.2% of the study population. 67.2% of the students age range from 18 to 20 years. Furthermore, nearly half of the participants (54.3%) studied in the medical college. Regarding family history of GIT tumors, 20.4% of participants had a positive family history of CRC (Table 1).

**Table 1:** Demographic and academic characteristics of the studied Egyptian university students

Socio-demographic variables		(*N* = 2090)	%
**Age (Years)**	**18–20 years**	1404	67.2
	**21–23 years**	580	27.8
	**24–26 years**	106	5.1
**Sex**	**Male**	572	25.2
	**Female**	1536	74.8
**Faculty**	**Medical**	1135	54.3
	**Non-medical**	955	45.7
**Academic year**	**First**	443	21.2
	**Second**	934	44.7
	**Third**	359	17.2
	**Fourth**	200	9.6
	**Fifth**	109	5.2
	**Sixth**	45	2.1
**Family history of GIT cell tumor**			426

### GIT: gastro-intestinal tumor

#### Knowledge regarding CRC

Analysis of the response rate of the knowledge about CRC among participants showed that the overall knowledge score was poor with a mean of 15.95 ± 6.5. Specific knowledge about CRC risk factors mean score was poor with a mean of 3.9 ± 1.95. The proportions of the students who approved that family medical history, smoking, and a high-fat diet as risk factors of CRC were 55.1%, 51.5%, and 48.8%, respectively. On the other hand, the mean knowledge score about CRC preventive factors was moderate with a mean of 3.08 ± 1.87. The majority of participants agreed that regular exercise, a diet rich in fiber, and maintaining a healthy body weight are considered preventive factors for CRC by 64.6%, 64.4%, and 61.4%, respectively. As regards knowledge about the warning signs, the mean score was poor with of mean of 3.53 ± 2.00, and the presence of blood in stool, change in bowel function, and abdominal pain were considered as warning signs for CRC by 67.7%, 61.8% and 60.2% of participants respectively. Knowledge about CRC screening methods score was poor with a mean of 5.4 ± 2.04 with more than 80% of the participants, considering the screening methods useful. 71.8% of students knew that CRC is a treatable disease if diagnosed early. Flexible sigmoidoscopy and CT were considered CRC screening procedures by 65.2% and 58.3% of participants, respectively (Table 2).

**Table 2:** Knowledge levels on CRC risk factors, warning signs, and screening methods among the studied participants

Knowledge items about CRC		Correct response *n* (%)			*P*-value
		Total (*N* = 2090)	Medical (*N* = 1135)	Non-medical (*N* = 955)	
**Knowledge about CRC risk factors**	**What is the CRC?**	1803(86.3%)	1056(93.0%)	747(78.2%)	< 0.0001*
	**Most risk age of CRC (> 50 years)**	580(27.8%)	332(29.3%)	186(19.5%)	< 0.0001*
	**Which of these constitute** **Risk factors of CRC? (smoking)**	1076(51.5%)	648(57.1%)	428(44.8%)	< 0.0001*
	**Which of these constitute** **risk factors of CRC? (High-fat diet)**	1020(48.8%)	600(52.9%)	420(44.0%)	< 0.0001*
	**Which of these constitute** **risk factors of CRC? (Old age)**	752(36.0%)	525(46.3%)	227(23.8%)	< 0.0001*
	**Which of these constitute** **risk factors of CRC? (Family medical history)**	1152(55.1%)	712(62.7%)	440(46.1%)	< 0.0001*
	**Which of these constitute** **risk factors of CRC? (Lack of physical activity)**	270(12.9%)	133(11.7%)	137(14.3%)	0.074
	**Which of these constitute** **risk factors of CRC? (Obesity)**	824(39.4%)	508(44.8%)	316(33.1%)	< 0.0001*
	**Which of these constitute** **risk factors of CRC? (Excessive intake of red or processed meat)**	754(36.1%)	446(39.3%)	308(32.3%)	0.001*
	***Mean ± SD score***	3.90 ± 1.95	4.37 ± 1.93	3.36 ± 1.82	< 0.0001*
**Knowledge about CRC preventive factors**	**Which of these constitute** **preventive factors of CRC? (Dietary rich in fiber diet)**	1346(64.4%)	817(72.0%)	529(55.4%)	< 0.0001*
	**Which of these constitute** **preventive factors of CRC? (Regular exercise)**	1351(64.6%)	814(71.7%)	537(56.2%)	< 0.0001*
	**Which of these constitute** **preventive factors of CRC? (Maintain a healthy body weight)**	1284(61.4%)	779(68.8%)	505(52.9%)	< 0.0001*
	**Which of these constitute** **preventive factors of CRC? (Stop smoking)**	1179(56.4%)	715(63.0%)	464(48.6%)	< 0.0001*
	**Which of these constitute** **preventive factors of CRC? (Keep drinking water)**	1279(61.2%)	766(67.5%)	513(53.7%)	< 0.0001*
	***Mean ± SD score***	3.08 ± 1.87	3.42 ± 1.75	2.66 ± 1.91	< 0.0001*
**Knowledge about CRC warning signs**	**Which of these constitute** **warning signs of CRC? (Blood in the stool)**	1414(67.7%)	881(77.6%)	533(55.8%)	< 0.0001*
	**Which of these constitute** **warning signs of CRC? (Abdominal pain)**	1259(60.2%)	707(62.3%)	552(57.8%)	< 0.0001*
	**Which of these constitute** **warning signs of CRC? (Anal pain)**	770(36.8%)	509(44.8%)	261(27.3%)	< 0.0001*
	**Which of these constitute** **warning signs of CRC? (Change in bowel function)**	1291(61.8%)	775(68.3%)	516(54.0%)	< 0.0001*
	**Which of these constitute** **warning signs of CRC? (Abdominal distention)**	1094(52.3%)	639(56.3%)	455(47.6%)	< 0.0001*
	**Which of these constitute** **warning signs of CRC? (Exhaustion)**	714(34.2%)	428(37.7%)	286(29.9%)	< 0.0001*
	**Which of these constitute** **warning signs of CRC? (Unexplained weight loss)**	855(40.9%)	552(48.6%)	303(31.7%)	< 0.0001*
	***Mean ± SD score***	3.53 ± 2.00	3.95 ± 1.96	3.04 ± 1.94	< 0.0001*
**Knowledge about CRC screening methods**	**Can CRC grow without obvious symptoms in the patient?**	1058(50.6%)	615(54.2%)	443(46.4%)	< 0.0001*
	**Is CRC a treatable disease if diagnosed early?**	1501(71.8%)	843(74.3%)	658(68.9%)	0.007*
	**Are there available screening methods to diagnose CRC early?**	1483(71.0%)	809(71.3%)	674(70.6%)	0.725
	**Are the screening methods useful?**	1707(81.7%)	915(83.8%)	756(79.2%)	0.006*
	**Which of the following procedures can be used in CRC screening? (colonoscopy)**	1293(61.9%)	842(74.2%)	451(47.2%)	< 0.0001*
	**Which of the following procedures can be used in CRC screening? (Fecal immunochemical test)**	406(19.4%)	260(22.9%)	146(15.3%)	< 0.0001*
	**Which of the following procedures can be used in CRC screening? (Fecal occult blood test)**	1032(49.4%)	622(54.8%)	410(42.9%)	< 0.0001*
	**Which of the following procedures can be used in CRC screening? (Stool DNA test)**	281(13.4%)	184(16.2%)	97(10.2%)	< 0.0001*
	**Which of the following procedures can be used in CRC screening? (Flexible sigmoidoscopy)**	1362(65.2%)	807(71.1%)	555(58.1%)	< 0.0001*
	**Which of the following procedures can be used in CRC screening? (CT colonography)**	1219(58.3%)	718(63.3%)	501(52.5%)	< 0.0001*
	***Mean ± SD score***	5.4 ± 2.04	5.85 ± 2.01	4.91 ± 1.96	< 0.0001*
**Mean ± SD)Total knowledge score (**		15.95 ± 6.5	17.61 ± 6.25	13.98 ± 6.22	0.000*

*Significant. -SD: Standard Deviation -CRC: Colorectal Cancer

Across overall and all specific knowledge domains, the medical college students had a highly significantly higher knowledge score than non-medical college students (*p* < 0.0001).

**Fig. 1:**
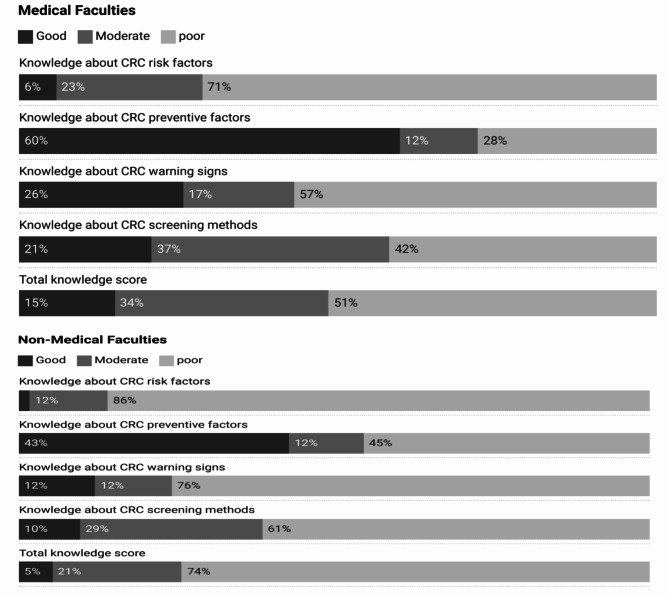
Knowledge Differences about CRC between Medical and Non-Medical Students

A comparison of knowledge score levels between students of medical and non-medical colleges revealed that only 6% of medical college students had good knowledge about CRC risk factors, 60% had good knowledge about CRC preventive factors, 26% knew CRC while 2% had good knowledge about CRC screening methods. Looking at the percentages for non-medical college students, only 2% of them had good knowledge about risk factors for CRC. The proportion of non-medical college students who had good knowledge about CRC preventive factors and medical warning signs was 12% for each Fig. 1.

### CRC: colorectal cancer

#### Practice and perceived barriers towards CRC screening modalities

**Fig. 2:**
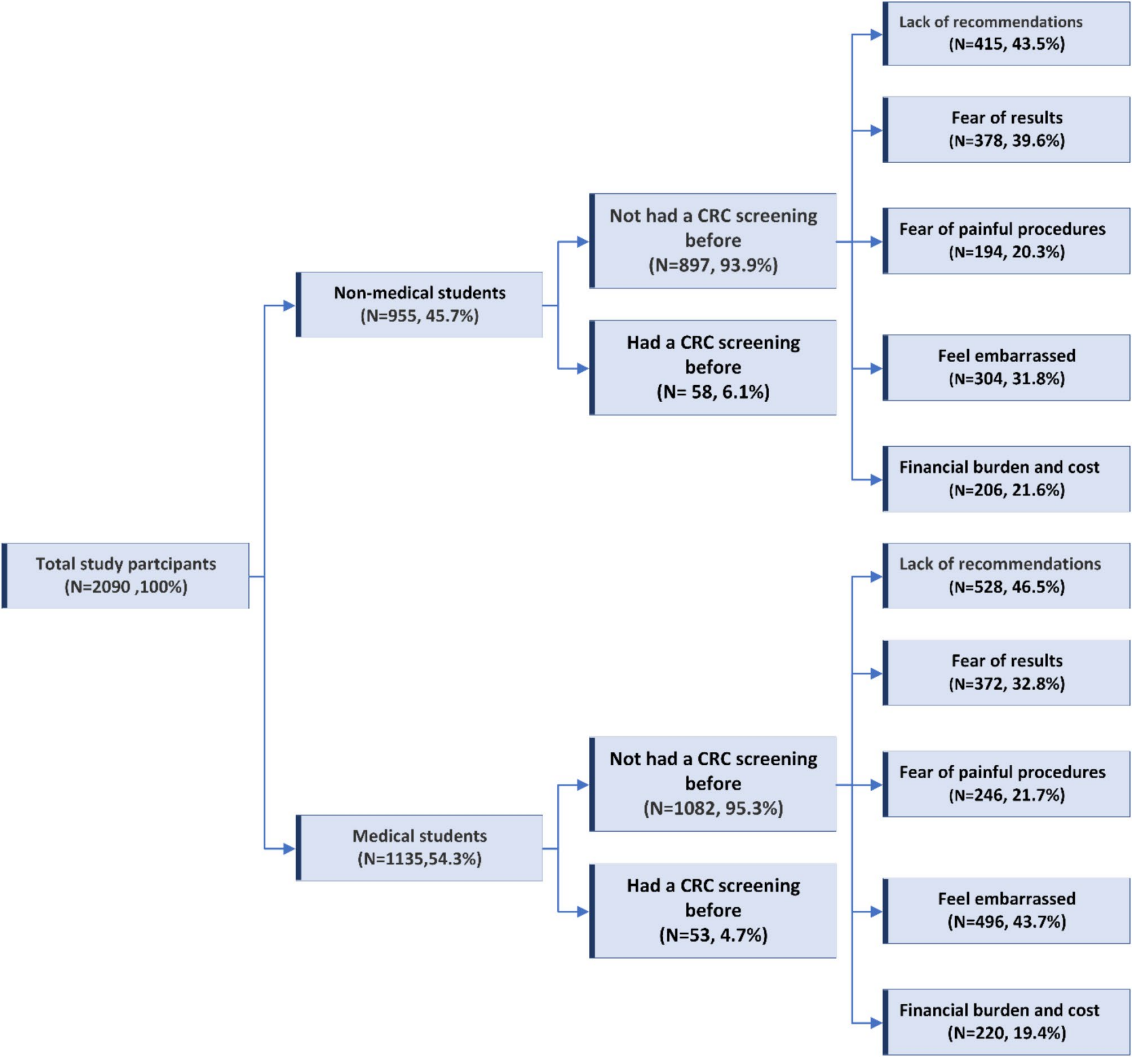
Flow Chart of Key Practice and Perceived Barriers Findings Towards CRC Screening between Medical and Non-Medical Students

Table 3; Fig. 2 illustrate the response rate of the practice and perceived barriers towards CRC screening modalities among the participants. Only 5.3% of the participants had undergone previous screening for CRC without a statistically significant difference between medical (4.7%) and non-medical college students (6.1%). Colonoscopy (76.5%) was the most used modality of CRC screening followed by CT colonography (23.5%). The most reported barrier for CRC screening was no available recommendation from the health care providers (45.1%) without a statistically significant difference between medical and non-medical college students. Feeling embarrassed (38.3%) was the second most reported barrier, with significantly higher proportions among medical college students (43.7%) as compared with non-medical college students (31.8%), (*p* < 0.0001). Also, fear of results was a significant barrier, with 35.9% of students reporting this as a reason for not having CRC screening. On the other hand, fear of results was recorded more frequently among nonmedical students than medical students, with a statistically significant difference (*p* = 0.001). Financial burden and cost were reported as a barrier by 20.4% of participants, while fear of painful procedures was reported by 21.1%. However, there were no significant differences between the two groups in terms of perceived barriers toward them.

**Table 3:** Practice and perceived barriers towards CRC screening among the studied participants

Practice and perceived barrier items	Correct response *n* (%)				
	Total (*N* = 2090)	Medical (*N* = 1135)	Non-medical (*N* = 955)	*P*-value	
**Have you had a CRC screening before?**	111(5.3%)	53(4.7%)	58(6.1%)	0.154	
**If yes**,** what type of screening did you do? (colonoscopy)**	85(76.5%)	38(71.6%)	47(81.0%)	0.105	
**If yes**,** what type of screening did you do? (CT colonography)**	26(23.5%)	15(28.4%)	11(19.0%)	0.650	
**If not**,** which of the following prevents you from taking it (Financial burden and cost)**	426(20.4%)	220(19.4%)	206(21.6%)	0.087	
**If not**,** which of the following prevents you from taking it (feel embarrassed)**	800(38.3%)	496(43.7%)	304(31.8%)	< 0.0001*	
**If not**,** which of the following prevents you from taking it (Fear of painful procedures)**	440(21.1%)	246(21.7%)	194(20.3%)	0.447	
**If not**,** which of the following prevents you from taking it (Fear of results)**	750(35.9%)	372(32.8%)	378(39.6%)	0.001*	
**If not**,** which of the following prevents you from taking it (No recommendation from the health care providers)**	943(45.1%)	528(46.5%)	415(43.5%)	0.161	

*Significant. -CRC: Colorectal Cancer

### CRC: colorectal cancer

#### Subgroup analysis of knowledge and practice levels regarding CRC

Table 4 presents a subgroup analysis of knowledge and practice levels regarding CRC across socio-demographic variables. Younger participants (18–20 years) exhibited the highest proportion of poor knowledge (64.4%) and practice (94.7% did not practice). Although there was no significant difference in knowledge levels between males and females (*p* = 0.564), males reported significantly higher practice rates compared to females (*p* < 0.0001). Academic year also influenced knowledge, with students in advanced years showing better knowledge levels (*p* < 0.0001). However, practice rates were not significant across academic years (*p* = 0.796). Participants with a family history of gastrointestinal tumors exhibited significantly higher practice rates (*p* = 0.012), although differences in knowledge were not statistically significant (*p* = 0.171).

**Table 4:** Subgroup analysis of knowledge and practice levels regarding CRC across Socio-Demographic variables

Socio-demographic variables		Knowledge			Practice	
		Good *N* = 221	Moderate *N* = 577	Poor *N* = 2090	Yes *N* = 111	No *N* = 1979
**Age (Years)**	**18–20 years**	124(8.8%)	376(26.8%)	904(64.4%)	74(5.3%)	1330(94.7%)
	**21–23 years**	86(14.8%)	170(29.3%)	324(55.9%)	26(4.5%)	554(95.5%)
	**24–26 years**	11(10.4%)	31(29.2%)	64(60.4%)	11(10.4%)	95(89.6%)
	***P-value***	0.001*			0.045*	
**Sex**	**Male**	57(10.8%)	136(25.8%)	334(63.4%)	48(9.1%)	479(90.9%)
	**Female**	164(10.5%)	441(28.2%)	958(61.3%)	63(4%)	1500(96%)
	***P-value***	0.564			< 0.0001*	
**Academic year**	**First**	25(5.6%)	83(18.7%)	335(75.6%)	48(9.1%)	479(90.9%)
	**Second**	63(17.5%)	101(28.1%)	195(54.3%)	27(6.1%)	416(93.9%)
	**Third**	83(8.9%)	283(30.3%)	568(60.8%)	22(6.1%)	337(93.9%)
	**Fourth**	21(19.3%)	41(37.6%)	47(43.1%)	45(4.8%)	889(95.2%)
	**Fifth**	24(12%)	53(26.5%)	123(61.5%)	6(5.5%)	103(94.5%)
	**Sixth**	5(9.2%)	16(36.3%)	24(54.5%)	10(5%)	190(95%)
	***P-value***	< 0.0001*			0.796	
**Family history of GIT cell tumor**	**Yes**	54(12.7%)	123(28.9%)	249(58.5%)	33(7.7%)	393(92.3%)
	***P-value***	0.171			0.012*	

*Significant. -GIT: Gastro-Intestinal Tumor

### Predictors of knowledge and practice regarding CRC

Table 5 presents possible predictors using a logistic regression model for knowledge & practice toward CRC among the participants. For knowledge predictors, the results show that students who had a medical education had significantly higher odds of having high knowledge scores (OR = 2.67, 95% CI: 1.91–3.71, *p* < 0.0001). Students who had ever heard about CRC had significantly higher odds of having high knowledge scores (OR = 6.80, 95% CI: 2.49–18.59, *p* < 0.0001). Moreover, being 21 to 23 years old is among the relevant predictors of high CRC knowledge (OR = 1.66, 95% CI: 1.22–2.25, *p* = 0.001). Gender and having relatives with CRC or being screened for CRC were not significant predictors of knowledge scores. For practice predictors, the results show that female students had significantly lower odds of having had CRC screening before (OR = 0.37, 95% CI: 0.25–0.56, *p* < 0.0001). Students who had relatives with CRC had significantly higher odds of having had CRC screening before (OR = 1.77, 95% CI: 1.15–2.73, *p* = 0.009). Age, education and ever hearing about CRC were not significant predictors of CRC screening practice.

**Table 5:** Predictors using logistic regression model for knowledge and practice towards CRC among the studied participants

Independent Variables		Knowledge Predictors (High Knowledge Scores ≥ 25) Odds ratio (95% C.I), *P* value	Practice of CRC Screening Before Odds ratio (95% C.I), *P* value
**Gender** **(Ref: Male)**	**Female**	0.98 (0.70–1.38), *p* = 0.942	0.37 (0.25–0.56), *p* < 0.0001 *
**Age Groups** **(Ref: 18–20 yrs)**	**21–23 yrs**	1.66 (1.22–2.25), *p* = 0.001 *	0.69 (0.43–1.11), *p* = 0.131
	**24–26 yrs**	1.08 (0.55–2.09), *p* = 0.820	1.65 (0.83–3.27), *p* = 0.148
**Education** **(Ref: Non-medical)**	**Medical**	2.67 (1.91–3.71), *p* < 0.0001 *	0.71 (0.48–1.05), *p* = 0.094
**Relatives with CRC**		1.34 (0.95–1.88), *p* = 0.088	1.77 (1.15–2.73), *p* = 0.009 *
**Ever Heard about CRC**		6.80 (2.49–18.59), *p* < 0.0001 *	1.82 (0.92–3.59), *p* = 0.083
**Screened for CRC**		0.048 (0.21–1.14), *p* = 0.098	-

*Significant. -CI: Confidence Interval -CRC: Colorectal Cancer

## Discussion

CRC is a significant public health concern with high impact, particularly when detected at later stages, making it crucial to understand the knowledge, practices, and barriers related to CRC screening among university students for effective early detection and prevention efforts. The results showed a great lack of knowledge about CRC, particularly its risk factors, especially among non-medical college students. Moreover, only a small proportion of students had undergone CRC screening (5.3%). The most reported barriers to CRC screening were the lack of recommendations from healthcare providers, feeling embarrassed, and fear of results. The current results suggest that medical education plays an important role in increasing the knowledge of CRC among students. Still, it is not sufficient to achieve a high level of knowledge.

Approximately half of the medical college students (51%) and three-quarters of the non-medical college students (71%) had poor overall knowledge about CRC. Specifically, the students had the poorest knowledge regarding the risk factors associated with CRC, followed by the disease’s warning signs, screening methods, and preventive factors. Only 6% of medical students had good knowledge of CRC risk factors, 60% knew preventive factors, 26% were aware of CRC, and 2% understood screening methods. Among non-medical students, 2% had good knowledge of risk factors, while 12% knew preventive factors and warning signs. When examining overall and specific knowledge domains, medical students significantly outperformed non-medical students in terms of their knowledge about different aspects of CRC. This indicates that medical college students generally possess a better understanding of CRC, which could be attributed to their specialized curriculum, clinical exposure, access to resources, peer learning, and mentoring from experienced healthcare professionals. These findings are consistent with other studies among adults in different countries, where more than half of their studied students had poor knowledge regarding CRC risk factors [25, 26]. This indicates that the gaps in CRC knowledge observed among the participant students are not unique to this cohort but reflect a broader phenomenon. In the context of university students, similar to our finding, Aga et al. reported better awareness of medical students in Saudi Arabia regarding different aspects of CRC than non-medical students [22]. In addition, another study among Saudi Arabian medical students has shown that more than half of them had inadequate knowledge about CRC [18]. On the other hands, another study in Jordon showed that university students exhibited good knowledge and awareness regarding CRC and its risk factors. In a study conducted in Pakistan by Hussain et al., it was revealed that the overall knowledge regarding CRC, encompassing its risk factors and symptoms, among participants stood at 44.8% [23]. While a majority of respondents demonstrated familiarity with CRC itself (66.6%) and its associated risk factors (50.9%), knowledge concerning warning signs was notably lacking, with only 40% awareness. This discrepancy could reflect cultural and regional differences and variations in the assessment tools [19].

Understanding and analyzing the predictors of CRC knowledge and screening practice are critical steps for communities that are trying to increase screening among priority populations [27]. Regarding knowledge predictors, the results show that students who had medical education and those who had previously heard about CRC had significantly higher odds of having high knowledge scores. Along the same line, different studies have explored the factors associated with CRC knowledge and found that factors such as education, prior awareness of CRC, and exposure to medical education or healthcare information are associated with higher knowledge scores [28–30]. In Saudi Arabia, Imran et al. highlighted that medical students displayed higher knowledge regarding CRC compared to non-medical students [31]. Several CRC screening modalities aimed at early detection have been developed, and there is a substantial body of evidence supporting the benefits of CRC screening [32]. Paradoxically, participation rates in screening programs were low worldwide, at an estimated 65% in the United States (US), and ranging from 1.9 to 54% across Europe had undergone previous CRC screening [33]. Among university students, Hussain et al., reported that 24.4% of participants had been involved in CRC screening [23]. In the present study, 5.3% reported that they had undergone screening for CRC. When considering university students, it is expected that the number of students who have undergone screening would be minimal. Generally, CRC screening isn’t recommended for this specific population to undergo routine CRC screening. Hence, it is reasonable to assume that the vast majority of these students would not meet the criteria for screening based on their age alone.

Family history can have a strong impact on screening behavior. Students who had relatives with gastrointestinal (GIT) tumors were more likely to have done CRC screening than those who did not. This may reflect their higher awareness of their risk and their proactive attitude towards their health. Previous literature supports this finding, as it has reported that individuals with affected relatives are often advised to start screening at a younger age [34].

This study reported that female participants were less likely to undergo CRC screening than males. This is consistent with the previous research by Lisa et al. (2011), who found women in the US and Canada had lower rates of CRC screening than men [35]. This disparity in screening rates for CRC compared to breast and cervical cancers may be attributed, in part, to the misperception among women and their physicians that CRC is primarily a “man’s disease.” However, it is worth noting that other studies have shown that women tend to have higher rates of preventive health behaviors, including CRC screenings [36]. This suggests that the observed pattern may be influenced by cultural or contextual factors that require further exploration in future research. Understanding these factors can provide valuable insights into the barriers and facilitators specific to female populations and help develop targeted interventions to address the disparities in CRC screening [37].

Understanding the barriers to CRC screening is crucial for developing effective screening programs aiming at increasing participation. Among the participants who had not undergone CRC screening, the most reported barrier was a lack of recommendations from health care providers. These findings are in accordance with the results of other studies where a lack of recommendations and a lack of a reminder system were reported as significant barriers [38, 39]. Moreover, the results of other studies supported the positive and direct relationship between healthcare provider recommendation and intention to undergo screening [40, 41]. Therefore, healthcare workers must communicate the benefits of CRC screening to their patients and encourage them to do it [42, 43].

Interestingly, in this study, feeling embarrassed was reported as a barrier to CRC screening significantly more often among medical college students compared to non-medical college students. This finding may be attributed to the higher level of knowledge about CRC screening practices and techniques among medical college students. The former students are more aware of potential discomfort or embarrassment associated with CRC screening procedures. In Egypt, the lack of easily accessible knowledge resources and awareness campaigns for the general population contributes to the existing barriers [12]. Similarly, Althobati et al. underscore that embarrassment and fear of diagnosis are prevalent barriers among medical students in Saudi Arabia, highlighting a broader issue that affects screening rates [18]. The lack of awareness about CRC and its screening methods, coupled with a shortage of trained healthcare providers, points to systemic issues that resonate with the barriers reported by medical students. On the other hand, fear of results was more frequently reported as a barrier among non-medical students than medical students. This discrepancy could be attributed to a lower proportion of non-medical college students who believe in the usefulness of screening tests compared to medical college students. In alignment with this observation, Hussain et al. pinpointed fear of a possible CRC diagnosis through screening, anxiety associated with screening procedures, and inadequate comprehension of CRC screening as predominant barriers to engaging in CRC screening practices among non-medical students in Pakistan [23]. It is noteworthy to mention that cultural perceptions in Egypt significantly shape the barriers to CRC screening, manifesting in feelings of embarrassment and fear regarding potential results. Bateman et al., in their study on Egyptian primary care physicians and specialists, highlighted how socioeconomic status and a lack of interest in preventive measures, influenced by the Egyptian cultural context, deter individuals from seeking screening [44]. The fear of receiving a cancer diagnosis, often perceived as a death sentence, further exacerbates this reluctance. Similar barriers have been identified in studies across the Middle East and North Africa region, indicating a broader cultural pattern that prioritizes treatment over prevention. Research on breast cancer screening in these areas reveals comparable issues, such as cultural stigma and fear, which hinder proactive health behaviors [45–47].

### Strength & limitations

#### Strengths

The strength of this study is its large sample size. Using a web-based questionnaire allowed for efficient data collection, reaching a wide range of participants. The study’s focus on university students is particularly relevant as they are a key target group for health education interventions. Additionally, the study assessed not only knowledge but also screening practices and perceived barriers, providing an overview of the factors influencing CRC screening behavior.

### Limitations

However, there are certain limitations to consider. Firstly, the study utilized a cross-sectional design, which limits the ability to establish causality or temporal relationships between variables. Secondly, data were collected using self-reported measures, which are subject to recall bias and social desirability bias. To mitigate recall bias in future research, mixed-methods approaches such as qualitative interviews or focus group discussions could be employed to validate self-reported data and provide deeper insights into students’ knowledge and perceptions of CRC. Additionally, longitudinal studies could help track changes in awareness and screening behaviors over time. Additionally, the study relied on voluntary participation. Hence, students who have a higher interest in health-related topics or those who had personal experiences with CRC may have been more likely to participate, leading to an overrepresentation of individuals with higher knowledge levels. To address self-selection bias, future research could implement random sampling techniques or actively recruit students from diverse academic disciplines and backgrounds to enhance representation. Moreover, unmeasured confounders such as socio-economic status, access to healthcare, or prior exposure to health campaigns were not assessed and could have influenced the study outcomes. Finally, the findings may not be generalizable to other populations outside of Egyptian university students or to those not engaged in academic settings, as the sample may not reflect the diversity of the broader population.

## Conclusion

In conclusion, this study underscores the concerning lack of knowledge and practice of CRC screening among university students, with notable disparities between medical and non-medical faculty students. The findings emphasize the need for focused educational interventions and awareness programs to bridge the knowledge gap, especially among non-medical university students, and promote early detection and prevention of CRC. Specific initiatives could include integrating CRC education into the university curriculum, utilizing interactive teaching methods, organizing student-centered screening campaigns, using peer-led educational initiatives, and leveraging social media campaigns to improve knowledge with a special focus on non-medical students. Collaborations with healthcare providers to deliver workshops and seminars can further enhance students’ understanding and engagement with CRC screening. Additionally, addressing barriers such as the absence of healthcare provider recommendations, feelings of embarrassment, and fear of results is crucial for improving screening rates. Ultimately, by addressing these issues, we can work towards reducing the burden of CRC and promoting a healthier future for all.

## Electronic supplementary material

Below is the link to the electronic supplementary material.

Supplementary Material 1

## Data Availability

Data is provided within the manuscript or supplementary information files.
