# Supplementary material for: A cross-sectional study on colorectal cancer screening knowledge and barriers among university students
Source: BMC Public Health. 2025 May 21;25:1871. doi: 10.1186/s12889-025-22510-z (PMC12093701; doi:10.1186/s12889-025-22510-z)
Supplement: Supplementary file 1 — Supplementary Material 1 [file 12889_2025_22510_MOESM1_ESM.pdf]

A Cross-Sectional Study on Colorectal Cancer Screening Knowledge and Barriers Among Egyptian University Students

**Do you agree to participate with us in this survey?**	Yes<br>No
**Socio-demographic characteristics**	
**Sex**	Male<br>Female
**Age**	
**Faculty**	
**Academic year**	First<br>Second<br>Third<br>Fourth<br>Fifth<br>Sixth
**Is there any Family history of GIT cell tumor**	Yes<br>No
**Knowledge about Colorectal Cancer**	
**Have you ever heard of colorectal cancer before?**	Yes<br>No
**What do you think is the most likely age to develop colon cancer?**	Less than 20 years<br>20-30 years<br>31-40 years old<br>41-50 years old<br>50 years or older*
**Which of the following constitute Risk factors of colorectal cancer? (*you can choose more than one*)**	Smoking<br>High fat diet<br>Aging<br>Medical history<br>Lack of physical activity<br>Obesity<br>Excessive intake of red or processed meat
**Which of the following constitute Preventive factors of colorectal cancer? (*you can choose more than one*)**	Dietary rich in fiber diet<br>Regular exercise<br>Maintain a healthy body weight<br>Stop smoking<br>Keep drinking water

**Which of the following constitute Warning signs of colorectal cancer? (*you can choose more than one*)**	Blood in the stool<br>Abdominal pain<br>Anal pain<br>Change in bowel function<br>Abdominal distention<br>Exhaustion<br>Unexplained weight loss
**Can colorectal cancer grow without obvious symptoms in the patient?**	Yes*<br>No
**Is colorectal cancer a treatable disease if diagnosed early?**	Yes*<br>No
**Are there available screening methods to diagnose colorectal cancer early?**	Yes*<br>No
**Are the screening methods useful?**	Yes*<br>No
**Which of the following procedures can be used in colorectal cancer screening? (*you can choose more than one*)**	colonoscopy<br>Fecal immunochemical test<br>Fecal occult blood test<br>Stool DNA test<br>Flexible sigmoidoscopy<br>CT colonography
**Practice & Perceived barriers regarding colorectal cancer**	
**Have you had a CRC screening before?**	Yes<br>No
**If yes, what type of screening did you do? (*you can choose more than one*)**	colonoscopy<br>Fecal immunochemical test<br>Fecal occult blood test<br>Stool DNA test<br>Flexible sigmoidoscopy<br>CT colonography
**If not, which of the following prevents you from taking it? (*you can choose more than one*)**	Financial burden and cost<br>feel embarrassed

Fear of painful procedures
Fear of results
no recommendation from the healthcare providers

***Correct Answer**

دراسة مقطعية حول معرفة وفهم فحص سرطان القولون والمستقيم والعوائق أمامه بين طلاب الجامعات المصرية

**الموافقة على المشاركة**	**هل توافق على المشاركة معنا في هذا الاستبيان؟**	**-نعم<br>-لا**
**الخصائص الديموغرافية**		
	**الجنس**	**-ذكر<br>-أنثى**
	**العمر**	***يرجى كتابة العمر بالأرقام***
	**الكلية**	***يرجى ذكر اسم الكلية***
	**السنة الدراسية**	**-الأولى<br>-الثانية<br>-الثالثة<br>-الرابعة<br>-الخامسة<br>-السادسة**
	**هل لديك تاريخ عائلي لأورام الجهاز الهضمي؟**	**-نعم<br>-لا**
**المعرفة حول سرطان القولون والمستقيم**		
	**هل سمعت عن سرطان القولون والمستقيم من قبل؟**	**-نعم<br>-لا**
	**ما هو العمر الأكثر عرضة للإصابة بسرطان القولون؟**	**-أقل من ٢٠ عامًا<br>-من ٢٠ إلى ٣٠ عامًا<br>-من ٣١ إلى ٤٠ عامًا<br>-من ٤١ إلى ٥٠ عامًا<br>-50 عامًا أو أكثر***
	**ما هي عوامل الخطر لسرطان القولون؟ (يمكنك اختيار أكثر من خيار)**	**-التدخين<br>-النظام الغذائي الغني بالدهون<br>-التقدم في العمر<br>-التاريخ الطبي<br>-قلة النشاط البدني<br>-السمنة<br>-الإكثار من اللحوم الحمراء أو المصنعة**
	**ما هي العوامل الوقائية من سرطان القولون؟ (يمكنك اختيار أكثر من خيار)**	**-التمارين الرياضية المنتظمة<br>-الحفاظ على وزن صحي**

	-الإقلاع عن التدخين<br>-شرب الماء بانتظام
ما هي العلامات التحذيرية لسرطان القولون؟ (يمكنك اختيار أكثر من خيار)	-وجود دم في البراز<br>-ألم في البطن<br>-ألم في الشرج<br>-تغير في وظيفة الأمعاء<br>-انتفاخ في البطن<br>-إرهاق<br>-فقدان وزن غير مبرر
هل يمكن أن ينمو سرطان القولون دون ظهور أعراض واضحة لدى المريض؟	-نعم *<br>-لا
هل يعتبر سرطان القولون مرضًا قابلًا للعلاج إذا تم تشخيصه مبكرًا؟	-نعم *<br>-لا
هل تتوفر طرق للفحص المبكر عن سرطان القولون؟	-نعم *<br>-لا
هل تعتبر طرق الفحص مفيدة؟	-نعم *<br>-لا
ما هي الطرق المتاحة لفحص سرطان القولون؟ (يمكنك اختيار أكثر من خيار)	-تنظير القولون<br>-اختبار الدم المناعي للبراز<br>-اختبار الدم الخفي في البراز<br>-اختبار الحمض النووي للبراز<br>-التنظير السيني<br>-التصوير المقطعي للقولون

الممارسات والعوائق المدركة

هل سبق وأن أجريت فحصًا لسرطان القولون؟	-نعم<br>-لا
إذا كانت الإجابة نعم، ما هو نوع الفحص الذي أجريته؟ (يمكنك اختيار أكثر من خيار)	-تنظير القولون<br>-اختبار الدم المناعي للبراز<br>-اختبار الدم الخفي في البراز<br>-اختبار الحمض النووي للبراز<br>-التنظير السيني<br>-التصوير المقطعي للقولون
إذا كانت الإجابة لا، ما الذي يمنعك من إجراء الفحص؟	-العبء المالي والتكلفة<br>-الشعور بالإحراج<br>-الخوف من الإجراءات المؤلمة<br>-الخوف من النتائج

-عدم وجود توصية من مقدمي الرعاية الصحية

*** الاجابة الصحيحة.**
